# Kinetics of acid-base parameters in conventional hemodialysis

**DOI:** 10.1590/1414-431X20187974

**Published:** 2018-12-10

**Authors:** J.R. Lugon, G.R.M. Pereira, J.P. Strogoff-de-Matos, A.J. Peixoto

**Affiliations:** 1 Divisão de Nefrologia, Departamento de Medicina, Faculdade de Medicina, Universidade Federal Fluminense, Niteroi, RJ, Brasil; 2 Curso de Pós-Graduação em Ciências Médicas, Faculdade de Medicina, Universidade Federal Fluminense, Niteroi, RJ, Brasil; 3 Section of Nephrology, Yale University School of Medicine, New Haven, CT, USA

**Keywords:** Hemodialysis, Renal replacement therapy, Kinetics, Adequacy of dialysis, Acid-base

## Abstract

Details about the acid-base changes in hemodialysis are scarce in the literature but are potentially relevant to adequate management of patients. We addressed the acid-base kinetics during hemodialysis and throughout the interdialytic period in a cross-sectional study of adults undergoing conventional hemodialysis. Samples for blood gas analysis were obtained from the arterial limb of the arteriovenous fistula before the first session of the week (HD1), immediately at the end of HD1, and on sequential collections at 15, 30, 45, 60, and 120 min post-HD1. Additional blood samples were collected after ∼20 h following the end of the first dialysis and immediately prior to the initiation of the second dialysis of the week. Thirty adult patients were analyzed (55±15 years, 50% men, 23% diabetic; dialysis vintage 69±53 months). Mean serum bicarbonate levels increased at the end of HD1 (22.3±2.7 mEq/L *vs* 17.5±2.3 mEq/L, P<0.001) and remained stable until 20 h after the end of the session. The mean values of pCO_2_ before HD1 were below reference and at 60 and 120 min post-HD1 were significantly lower than at the start (31.3±2.7 mmHg and 30.9±3.7 mmHg *vs* 34.3±4.1 mmHg, P=0.041 and P=0.010, respectively). The only point of collection in which mean values of pCO_2_ were above 35 mmHg was 20 h post-dialysis. Serum bicarbonate levels remained stable for at least 20 h after the dialysis sessions, a finding that may have therapeutic implications. During dialysis, the respiratory response for correction of metabolic acidosis (i.e., pCO_2_ elevation) was impaired.

## Introduction

Hemodialysis patients have a high mortality rate, with cardiovascular disease (CVD) accounting for about 50% of the fatalities (1 [Bibr B02]). Compared with the general population, patients with end-stage renal disease (ESRD) have an age-adjusted CVD mortality rate that is 15 to 30 times higher (1–3). A number of studies indicate that the presence of metabolic acidosis is associated with high mortality (4–7). On the other hand, metabolic alkalosis has also been associated with increased mortality in dialysis in some settings (7,8).

Conventional hemodialysis treatment may not be sufficient for adequate control of acidosis in ESRD patients (9), and complementary interventions may be required. Elevation of bicarbonate concentration in the dialysate would be an acceptable alternative for better management of metabolic acidosis in this setting but carries a potential increase in risk of mortality (5 [Bibr B06]). In addition, customization of dialysate composition is not routinely feasible in many dialysis centers. The other option, oral bicarbonate supplementation, may represent a further burden for ESRD patients already exposed to polypharmacy, besides representing an added risk of sodium overload.

Detailed understanding of acid-base balance in the intra- and interdialytic periods is essential for the management of acid-base disorders in hemodialysis patients. To that effect, the available literature is scarce and dates from a time when acetate was the predominant dialysate buffer and patients were treated using non-conventional dialysis schedules (10). To our knowledge, the present study is the first to provide a detailed description of the kinetics of the full set of arterial blood gas-based acid-base parameters in patients receiving conventional hemodialysis treatments.

## Material and Methods

This was an observational cross-sectional study conducted in patients undergoing conventional thrice-weekly chronic maintenance hemodialysis for at least three months at a single dialysis center in Niteroi, Rio de Janeiro, Brazil. A survey performed at the beginning of the study showed that the 5-year survival rate of incident patients of the center was 67%. Of 184 patients, we selected 32 patients by convenience sampling, who were randomly chosen through a computer-generated lottery. The following exclusion criteria were applied: age less than 20 years, use of catheter as vascular access, known chronic lung disease, or active hepatitis B, hepatitis C, or HIV. The Ethics Committee of the Universidade Federal Fluminense School of Medicine approved the study and all patients provided signed informed consent. The study conformed to the principles of the Declaration of Helsinki.

### Procedures

Dialysis was performed using 4008S hemodialysis machines (Fresenius Medical Care, Germany), and high-flux polyamix dialyzers (Polyflux 2.1^®^, Gambro, Germany), which were reprocessed with peracetic acid as the sterilant by an automated system for a maximum of 20 times in accordance to Brazilian regulations. Fresenius Medical Care (Brazil) manufactured all the dialysates used. Characteristics of the dialysis treatment are listed in Table 1. Blood samples were collected before the first session of the week, immediately after this session, and at time intervals of 15, 30, 45, 60, and 120 min post-hemodialysis. Additional blood samples were collected on the non-dialysis days (about 20 h after the end of dialysis) and immediately before the second session of the week. Before the dialysis sessions, samples were collected directly from the indwelling needle used to puncture the arterial limb of the arteriovenous fistula after discarding the blood corresponding to the priming volume of the device. Pre-dialysis samples were placed on ice and taken immediately to the laboratory. Syringes containing the first five post-dialysis samples (until the 60th min sample) were kept on ice and taken to the laboratory immediately after the end of the collections. The other samples (at 120 min and 20 h post-dialysis sessions) were placed on ice and taken immediately to the laboratory. None of the samples was refrigerated for more than 70 min until blood gas analysis.

**Table 1:** Characteristics of dialysis treatment.

Duration of sessions, h	4
Single pool Kt/V*	1.56±0.08^a^
Dialyzer use at data collection	8±6
Blood flow, mL/min	350 (200-400)^b^
Dialysate flow, mL/min	500
Dialysate composition after dilution, n (%)	
Sodium 138 mEq/L	30 (100)
Calcium, n (%)	
2.5 mEq/L	7 (23.3)
3.0 mEq/L	22 (73.3)
3.5 mEq/L	1 (3.4)
Magnesium 1.0 mEq/L, n (%)	30 (100)
Acetate 4.0 mEq/L, n (%)	30 (100)
Bicarbonate 31.4 mEq/L, n (%)	30 (100)
Glucose 100 mg/dL, n (%)	30 (100)

*Data refer to routine measurements of the last 3 months of each patient before enrollment (post-dialysis sample was collected by the slow blood flow method following KDIGO recommendations). ^a^Mean±SD; ^b^Median (range).

### Parameters and estimates

We collected all clinical data and routine laboratory tests from patients' charts. Blood pressure values were taken as the mean of pre-dialysis measurements of the last three dialysis sessions before enrollment. Routine laboratory tests were extracted from the patient's chart and calculated as the mean of the last three available values. Serum albumin was measured by the green bromocresol method. Acid-base values were all derived from the blood gas analysis performed as a specific study procedure (ABL5, Radiometer Medical A/S, Denmark).

Normalized protein nitrogen appearance (nPNA) was calculated by the formula: BUN / (36.3 + 5.48 × Kt/V + (53.5/Kt/V)) + 0.168, in which Kt/V was obtained by: –Ln (post BUN/pre BUN – 0.008 × t) + (4 – (3.5 × post BUN/pre BUN)) x UF/W (11). The respiratory response to metabolic acidosis and metabolic alkalosis were estimated by the formulas Expected pCO_2_ = 1.2 × current HCO_3_^-^ + 11.2 (12) and Expected pCO_2_ = 40 + ((current HCO_3_^–^ –24) × 0.7), respectively (13).

### Statistical analysis

Continuous variables are reported as mean±SD or median and internal quartiles, as appropriate. Categorical variables are reported as frequencies. Differences between continuous dependent variables were always tested by Friedman's ANOVA followed by the Tukey's test. Mann-Whitney tests were used to compare two independent variables. Statistical analyses were performed using SPSS, version 18.0 for Windows (IBM SPSS, USA).

## Results

**Figure 1:**
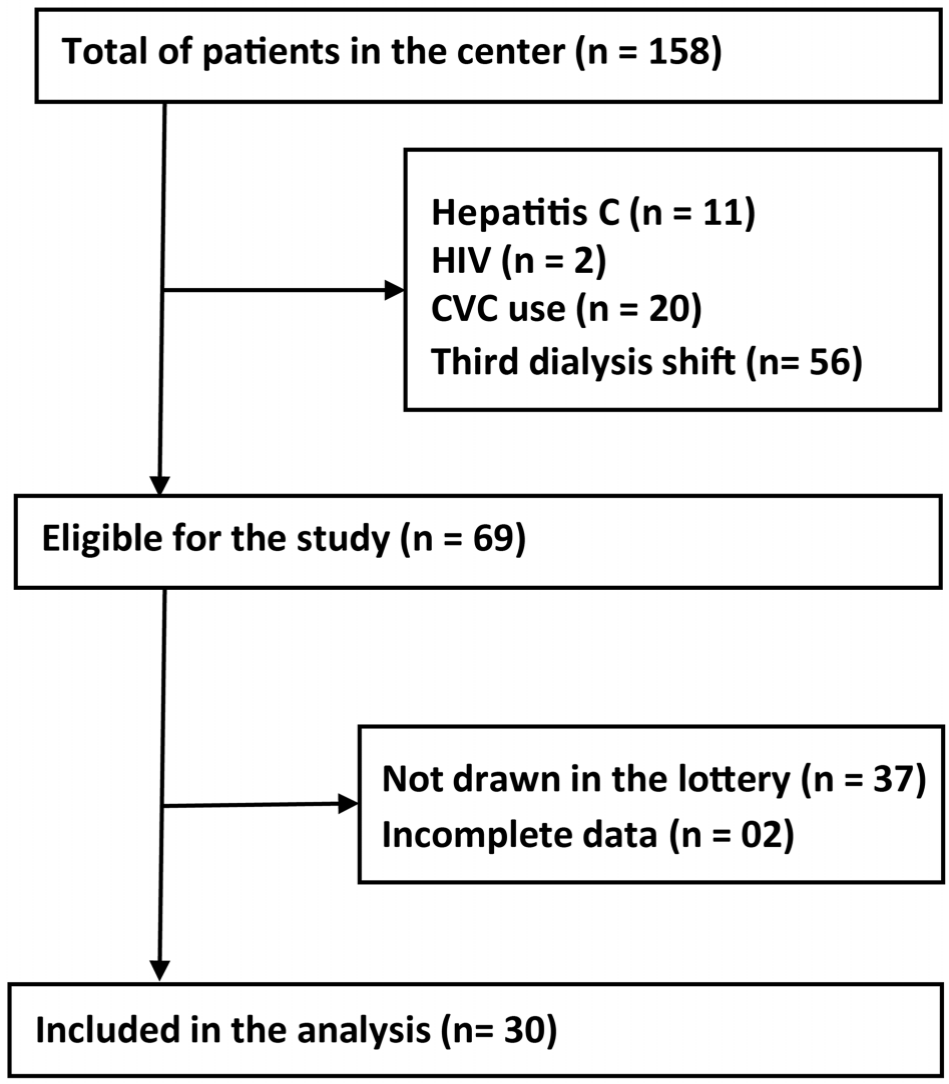
Selection of study participants. HIV: Human immunodeficiency virus; CVC: central venous catheter.

Thirty-two subjects were recruited for the study; two of them were excluded from analysis due to incomplete data, thus leaving 30 subjects for analysis (Figure 1). Clinical and laboratory features are presented in Table 2.

**Table 2:** General characteristics of patients.

Male gender, n (%)	15(50)
Age, years	55±15
Race, n (%)	
White	10 (33.3)
Non-White	20 (66.7)
Body weight, kg	
Pre-HD1/	67.9±14.1
Post-HD1	65.4±14.1
Body mass index, kg/m^2^	
Male	23.2±4.4
Female	24.8±5.3
Primary renal disease, n (%)	
Diabetes mellitus	7 (23.3)
Hypertension	17 (56.7)
Chronic glomerulonephritis	4 (13.3)
Other	2 (6.7)
Pre-dialysis blood pressure, mmHg*	
Systolic	129±12
Diastolic	80±8
Interdialytic weight gain, kg	2.52±2.1
Dialysis vintage, months	69±53
Presence of residual urine volume, n (%) of patients	11 (36.7)
Urine volume of the interval, mL^φ^	486±370
BUN, mg/dL^δ^	
Pre-HD1	61.5±16.9
Post-HD1	18.0±6.5
Midweek pre-dialysis BUN	61±15
Serum albumin, g/dL^δ^	3.9±0.4
Serum calcium, mg/dL^δ^	8.8±0.4
Serum phosphorus, mg/dL^δ^	4.7±0.9
Intact parathormone, pg/mL^δ^	159 (82-458)
Hemoglobin, g/dL^δ^	11.5±1.2
Normalized protein nitrogen appearance, g/kg per day^δ^	0.93±0.18

Data are reported as means±SD or median (IQR). HD1: First dialysis session of the week; *Data refer to measurements for the last 3 sessions of each patient before enrollment; ^φ^Data refer to a 44-h collection and only pertain to the 11 patients who had residual urine volume; ^δ^Data refer to routine measurements calculated as the mean of the last three available results in the chart for each patient.

None of the patients had hypotensive episodes (systolic blood pressure <100 mmHg) during the studied dialysis sessions. The ranges of pre-dialysis total CO_2_ (tCO_2_) for the midweek dialysis sessions are presented in Table 3.

**Table 3:** Range of pre-dialysis total CO_2_ (tCO_2_) of the midweek dialysis session in the studied sample.

tCO_2_ (mEq/L)	n	%
<18	9	30.0
18-<20	6	20.0
20-<22	10	33.3
22-<24	3	10.0
24-<26	1	3.3
≥26	1	3.3
Total	30	100.0

**Figure 2:**
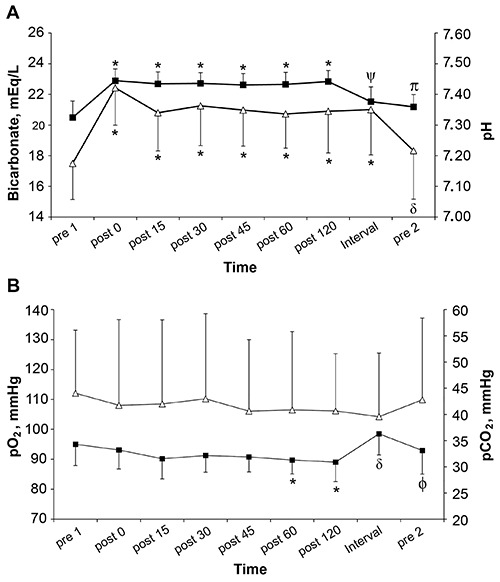
*A*, Kinetics of bicarbonate (open triangles) and pH (closed squares), and *B,* of pO_2_ (open triangles) and pCO2 (closed squares). Data are reported as means±SD. Pre: immediately before the dialysis session (numbers 1 and 2 refer to the 1st and 2nd sessions of the week); post: after the 1st dialysis session of the week (numbers refer to min after the end of the session); interval: 20 h after the end of the first session of dialysis of the week. P<0.050: *vs pre1; ^Ψ^ *vs* all previous measures; ^π^ *vs* all previous measures except interval; ^δ^ *vs* all previous measures except pre1; ^Φ^ *vs* interval (ANOVA followed by the Tukey's test).

The kinetics of measured acid-base parameters throughout the nine sampling time-points are reported in Figure 2. Mean serum bicarbonate levels increased at the end of the first dialysis session of the week (22.3±2.7 mEq/L *vs* 17.5±2.3 mEq/L pre-dialysis, P<0.001) and tended to show a slight decrease of approximately 7% at 15 min post-dialysis, remaining stable until 20 h after the end of the session (mid-point interdialytic sample). Immediately prior to the second dialysis session of the week, mean serum bicarbonate was not statistically different from that before the first dialysis of the week (18.3±3.2 mEq/L *vs* 17.5±2.3 mEq/L, P=0.947). Mean values for pH prior to and immediately after the first dialysis session exhibited the same trends as for bicarbonate, but the reduction toward lower levels was already present in the mid-point interdialytic sample. Again, mean values immediately prior to the second dialysis of the week were in the lower range but somewhat higher than those seen before the first dialysis of the week (7.360±0.040 *vs* 7.325±0.054, P=0.032).

Mean values of pO_2_ remained stable and above 100 mmHg (with oxygen saturation above 97%) during all measurement points. Mean pCO_2_ values before the first dialysis of the week were below reference and at the end of the dialysis session tended to be lower than at the beginning but did not reach statistical significance (33.2±3.6 mmHg *vs* 34.3±4.1 mmHg, P=0.960). Of note, values at 60 min (31.3±2.7 mmHg) and 120 min (30.9±3.7 mmHg) were statistically lower than at pre-dialysis (P=0.041 and P=0.010, respectively). The only point of collection in which mean values of pCO_2_ were above 35 mmHg was 20 h post-dialysis. Prior to the second dialysis session of the week, the mean pCO_2_ was again below the normal values and comparable to values found before the first dialysis of the week (33.1±4.5 mmHg *vs* 34.3±4.1 mmHg, P=0.936).

The detailed values of pH, serum bicarbonate, and blood pCO_2_ for each patient at each time-point are presented in the Supplementary Figures S1-S3 and show that the behavior of the variables was very homogeneous.

Forty-seven percent of our patients were using sevelamer hydrochloride, 30% calcium carbonate and 7% calcium acetate. The pre-dialysis pH and bicarbonate levels did not differ from patients receiving or not receiving sevelamer hydrochloride (7.31±0.07 *vs* 7.32±0.06, P=0.552 and 16.8±2.4 mEq/L *vs* 17.4±2.7 mEq/L, P=0.668, respectively).

## Discussion

In this study, we presented a detailed analysis of the behavior of the full set of arterial blood gas-based acid-base parameters during the interdialytic period in stable chronic hemodialysis patients with clinical characteristics similar to the typical hemodialysis population in Brazil (14). To our knowledge, this is the first such detailed report in patients receiving chronic maintenance hemodialysis with contemporary equipment and general prescription. As can be judged by mean body mass index, mean serum levels of albumin, calcium, phosphorus, iPTH, and hemoglobin, and by mean urea reduction ratio, our subjects received dialysis treatment well within the standards of adequacy. However, the mean nPNA indicated a protein intake on the low range of values usually reported for hemodialysis patients (15–17), possibly due to economic issues to afford protein-rich foods. Additionally, it is apparent that the dialysate bicarbonate levels used by our center were slightly lower than those reported by most centers worldwide. However, this usual concentration in Brazil is similar to the most commonly used in Germany and higher than those in Japan (5). In a recent multinational study, the used dialysate bicarbonate levels were grouped as values ≤32 mEq/L, 33–37 mEq/L, and ≥38 mEq/L (5). About 25% of centers were on the high side of the range with only ∼15% of centers on the low side. The relatively low dialysate bicarbonate levels used in our center can in part explain the lower mean pre-dialysis serum bicarbonate in a midweek session of our sample (19.3 mEq/L) compared to global data reported in two studies, 21.9 mEq/L (4) and 22.9 mEq/L (5). The difference persisted even when the comparison was made with the mean values of serum bicarbonate (21.4 mEq/L) of the centers that also use low dialysate bicarbonate (5). Our patients were approximately ten years younger and none of our patients was under oral bicarbonate therapy in contrast to 10.9% in that cohort, variables that may have contributed to the observed difference in the mean serum bicarbonate of the studied groups.

The present study was not designed to address the issue of what is the adequate pre-dialysis serum level of bicarbonate. The Disease Outcome Quality Initiative (DOQI) recommendation of a pre-dialysis serum tCO_2_ level ≥22mEq/L without specifying whether before the first or second session of the week dates from the year 2000 and was not reviewed since then (11). Our values before the second dialysis sessions of the week were below the DOQI recommendation in 83%. Of note, only one patient had a pre-dialysis serum tCO_2_ level slightly higher than 26 mEq/L, allowing us to conclude that none of our patients had unsafe levels of metabolic alkalosis pointed out in previous studies (8,9,18,19). The issue of the target bicarbonate levels remains a matter of debate; in a large study (4), values ≥18mEq/L were thought to be closer to an ideal target, also supported by a recent review (20). In the present study, 30% of the serum tCO_2_ concentrations before the second dialysis session of the week remained ≤18 mEq/L. The figure is higher than the 16% reported in a global study (4) reflecting a more acidotic state of our cohort, as previously discussed. Interestingly, our patients maintained adequate nutrition (mean serum albumin 3.9 g/dL), despite the relatively low bicarbonate and low nPNA. Considering that the functioning of biological machinery is mainly influenced by changes in (H^+^), not (HCO_3_^-^), we wondered if the maintenance of serum pH well within a safe range at every studied point along the week could in part account for these findings.

An integrative view of findings at the start of the first dialysis of the week showed slight acidemia (mean pH 7.32±0.05), mean bicarbonate concentration of 17.5±2.3 mEq/L, and an adequate respiratory response for the level of chronic metabolic acidosis (mean difference between measured and expected pCO_2_ values, 2.1 mEq/L). These findings are consistent with a recent study in which these types of pre-dialysis acid-base alterations were reported among the most frequent ones (21).

At the end of the first dialysis session of the week, serum bicarbonate was 22.3±2.7 mEq/L, which is 9 mEq/L below the dialysate bicarbonate concentration. At this point of collection, pH was in the normal range (7.44±0.04), but pCO_2_ was inappropriately low (mean difference between measured and expected pCO_2_ values, –5.6 mEq/L) and continued to decrease until 120 min after the end of the dialysis session.

The lag in respiratory adjustment after correction of metabolic acidosis in a hemodialysis session was reported in the early days of dialysis (22) and is known to occur in hemodialysis sessions performed with either bicarbonate (22,23) or acetate-based dialysate (10,24,25). Several explanations for the phenomenon were raised, including acid-base disequilibrium between either the cell compartments (22) or the blood-brain barrier (26), chronic change in the brain threshold for (H^+^) (27), or blood-brain osmotic disequilibrium (24). Intriguingly, this last study reported adequate respiratory response when urea was added to the dialysate to match the serum urea concentration on the day prior to dialysis implicating hypo-osmolality as a possible subjacent cause for hyperventilation.

Another potential explanation when trying to account for hyperventilation in hemodialysis is hypoxia. When hemodialysis was performed with cellulose membranes, episodic hypoxia could occur due to pulmonary sequestration of leukocytes linked to activation of the alternate pathway of the complement system (28). However, our patients were treated with synthetic membranes and their pO_2_ and O_2_ saturation remained stable throughout the procedure. Finally, when trying to account for the persistence of low pCO_2_ despite correction of the metabolic acidosis in hemodialysis, the issue of potential CO_2_ loss through the dialyzer membrane needs to be addressed. In a previous publication, contrary to expectation, the pCO_2_ of the efferent bloodline was higher than that of the afferent line (29). The authors reasoned that this finding was caused by the gain of bicarbonate when blood crosses the dialyzer. Consistent with the present study, the authors also reported hyperventilation and increased CO_2_ excretion during the procedure. Regardless of cause, persistent hyperventilation in hemodialysis may have clinical relevance: the initial metabolic acidosis may be converted to a mixed metabolic and respiratory alkalosis at the end of dialysis and aggravate electrolyte imbalance predisposing to cardiac arrhythmias. The magnitude of this potential complication is conceivably higher when a high dialysate bicarbonate concentration is used. It is tempting to think that the recently reported 8% increase in overall mortality of dialysis patients for each 4 mEq/L elevation in dialysate bicarbonate concentration (5) may be, in part, related to this phenomenon.

Of note, a trend for a reduction of 7% in the serum bicarbonate concentration was observed in the first 15 min post-dialysis perhaps due to equilibration between compartments. From then on, bicarbonate levels remained stable until patients left, 2 h after the end of the dialysis session.

At the interval visit, in comparison to the sample collected 120 min after the previous dialysis session, mean pH was lower (but still in the normal range), mean pCO_2_ was higher, and mean bicarbonate concentration was unchanged, allowing us to conclude that the reduction in pH at this point seemed to be entirely accounted for by pCO_2_ normalization. The intriguing persistence of an adequate bicarbonate concentration in the day after the dialysis session was already reported at a time when acetate was the standard buffer dialysate and authors resorted to low rate of acetate metabolism to explain the finding (10). Of interest, serum bicarbonate was measured during a mid-week dialytic interval in nine patients dialyzed for 4 h using a dialysate bicarbonate concentration of 35 mEq/L (30). The mean reduction rate of serum bicarbonate level in the first 20 h post dialysis was 0.065 mEq/L per hour in contrast to 0.121 mEq/L per hour in the remaining 24 h. Unfortunately, the authors did not report the dialysate acetate concentration, which could be of value in interpreting the finding, and other acid-base parameters were not measured in that study. The information about acetate metabolism in hemodialysis is scant and derived from studies with acetate-based dialysate. By that time, authors reported a fast rate of conversion of the compound in the muscle (31–33), with one study emphasizing that a small fraction of the dialysis population was unable to metabolize acetate properly (32). The driving forces for acetate metabolism rate are unknown, but the concentration of the compound itself and the level of metabolic acidosis aggravated by the loss of bicarbonate to the dialysate are acceptable candidates under that circumstance. Now that bicarbonate buffered dialysate is the gold standard in clinical practice, the acetate concentration in the dialysate is very low and the level of serum bicarbonate during the dialysis session is kept close to or even higher than the normal range. In this setting, the rate of acetate metabolism can be conceivably slower than previously reported. One could wonder if the small amount of acetate present in the bicarbonate buffered dialysate would be enough to keep the serum bicarbonate concentration at a normal range 20 h after the dialysis session. The daily acid generation in hemodialysis patients seems to be lower than in the general population, calculated at about 0.5 mEq/Kg (34). If a volume of distribution for acetate of 40% of body weight were assumed, the amount of bicarbonate generated from an acetate serum level close to 4 mEq/L would potentially exceed the amount required for that task.

Finally, it should be commented that the low nPNA of our patients might have contributed to the stability of the serum bicarbonate until 20 h post-dialysis. Irrespective of cause, the persistence of adequate levels of serum bicarbonate may have therapeutic implications: when deciding for oral bicarbonate replacement, our findings suggest that administration could be postponed for at least 20 h after the end of the dialysis session.

The serum bicarbonate levels started to fall sometime between 20 and 44 h after the first dialysis. At the beginning of the second dialysis session of the week, values for serum pH, bicarbonate, and pCO_2_ were close to the ones before the first dialysis of the week but with a slightly lower deviation from the reference values. We did not measure the behavior of these variables following the second hemodialysis session and the rest of the week.

Our study presented some limitations. The number of enrolled patients was relatively small, the dialysate bicarbonate concentration used was in the lower range, the frequency of metabolic acidosis in our patients was high, and the nPNA of our patients was in the low range, limiting the generalizability of the findings. In addition, other electrolytes that could help to better understand the totality of acid base balance were not measured. Nevertheless, these limitations do not detract from the valuable mapping of pH, bicarbonate, and pCO_2_.

In summary, the present study provides data on the kinetics of acid base parameters in hemodialysis patients undergoing bicarbonate buffered chronic hemodialysis. Findings confirmed the paradoxical respiratory response of hemodialysis patients for correction of their metabolic acidosis that can, in settings of high dialysate bicarbonate use, result in a mixed alkalosis state at the end of a dialysis session. The study also showed that serum bicarbonate concentration remained unchanged at least until 20 h after the end of a dialysis session, a finding with potential therapeutic implications.

## Supplementary Material

Click here to view [pdf].
